# Selection signatures in tropical cattle are enriched for promoter and coding regions and reveal missense mutations in the damage response gene *HELB*

**DOI:** 10.1186/s12711-020-00546-6

**Published:** 2020-05-27

**Authors:** Marina Naval-Sánchez, Laercio R. Porto-Neto, Diercles F. Cardoso, Ben J. Hayes, Hans D. Daetwyler, James Kijas, Antonio Reverter

**Affiliations:** 1 CSIRO Agriculture & Food, 306 Carmody Rd., St. Lucia, Brisbane, QLD 4067 Australia; 2 Department of Animal Science, School of Agricultural and Veterinarian Sciences, Sao Paulo State University (UNESP), Jaboticabal, SP Brazil; 3 Queensland Alliance for Agriculture and Food Innovation, The University of Queensland, St. Lucia, QLD 4067 Australia; 4 Agriculture Victoria, AgriBio, Centre for AgriBioscience, Bundoora, VIC 3083 Australia; 5 School of Applied Systems Biology, La Trobe University, Bundoora, VIC 3083 Australia; 6 Present Address: Institute of Molecular Biosciences, The University of Queensland, 306 Carmody Road, St. Lucia, Brisbane, QLD 4067 Australia; 7 Present Address: Centre for Genetic Improvement of Livestock, University of Guelph, 50 Stone Road East, Guelph, ON N1G2W1 Canada

## Abstract

**Background:**

Distinct domestication events, adaptation to different climatic zones, and divergent selection in productive traits have shaped the genomic differences between taurine and indicine cattle. In this study, we assessed the impact of artificial selection and environmental adaptation by comparing whole-genome sequences from European taurine and Asian indicine breeds and from African cattle. Next, we studied the impact of divergent selection by exploiting predicted and experimental functional annotation of the bovine genome.

**Results:**

We identified selective sweeps in beef cattle taurine and indicine populations, including a 430-kb selective sweep on indicine cattle chromosome 5 that is located between 47,670,001 and 48,100,000 bp and spans five genes, i.e. *HELB*, *IRAK3*, *ENSBTAG00000026993*, *GRIP1* and part of *HMGA2*. Regions under selection in indicine cattle display significant enrichment for promoters and coding genes. At the nucleotide level, sites that show a strong divergence in allele frequency between European taurine and Asian indicine are enriched for the same functional categories. We identified nine single nucleotide polymorphisms (SNPs) in coding regions that are fixed for different alleles between subspecies, eight of which were located within the *DNA helicase B* (*HELB*) gene. By mining information from the 1000 Bull Genomes Project, we found that *HELB* carries mutations that are specific to indicine cattle but also found in taurine cattle, which are known to have been subject to indicine introgression from breeds, such as N’Dama, Anatolian Red, Marchigiana, Chianina, and Piedmontese. Based on in-house genome sequences, we proved that mutations in *HELB* segregate independently of the copy number variation *HMGA2*-CNV, which is located in the same region.

**Conclusions:**

Major genomic sequence differences between *Bos taurus* and *Bos indicus* are enriched for promoter and coding regions. We identified a 430-kb selective sweep in Asian indicine cattle located on chromosome 5, which carries SNPs that are fixed in indicine populations and located in the coding sequences of the *HELB* gene. *HELB* is involved in the response to DNA damage including exposure to ultra-violet light and is associated with reproductive traits and yearling weight in tropical cattle. Thus, *HELB* likely contributed to the adaptation of tropical cattle to their harsh environment.

## Background

The domestication of wild aurochs (*Bos primigenous*) in two distinct locations, in the Middle East (~ 10,000 years ago) and the Indian subcontinent (~ 8000), resulted in the separate evolution of two cattle lineages and in divergences between the genomes of taurine (*Bos primigenous taurus*) and indicine (*Bos primigenous indicus*) cattle. In general, they occupy distinct geographic and climatic locations worldwide [1, 2]. Taurine cattle are mostly found in temperate environments, whereas indicine breeds are highly adapted to environments with constant high temperatures [3]. Besides adaptation to heat, other environmental adaptation traits such as disease and parasite resistance, and differences in human herd management and selection processes have driven different patterns of genomic variation between these cattle sub-species. This offers the opportunity to identify genes that are involved in adaptation, within the genetic context of a single species. The identification of genomic regions impacted both by human selection and climate adaptation will help understand how changes at the genome level modulate changes in phenotype, which holds high promises to improve animal breeding processes for production, health, and welfare [4]. Previous analyses using single nucleotide polymorphism (SNP) arrays [5–13] and whole-genome sequences [14, 15] have identified candidate regions and potential genes under selection in various cattle breeds. However, compared to other domestic species for which selection is known to impact mostly conserved elements, transcription start sites or regulatory regions [16–18], to our knowledge, there has been no effort to understand the impact of selection at the functional-genomic level in cattle. To date, the lack of functional genomic information has limited the attempts to analyze the impact of selection and evolutionary divergence in cattle and in other livestock species.

Recently, an international effort entitled ‘The Functional Annotation of Animal Genomes’ (FAANG; https://www.animalgenome.org/community/FAANG/index) has been launched and aims at addressing the above issue by experimentally identifying regulatory regions in the genomes of many tissues and at several stages of development [19]. Meanwhile, our group has recently provided a first draft of cattle and sheep functional regulatory regions based on the identification of orthologous regulatory regions [18, 20] in other species from the human and mouse ENCODE [21, 22] and RoadMap consortia [23].

In this work, our objectives were to investigate genomic differences between *Bos taurus* and *Bos indicus* in the European versus Indian subcontinents and between African taurine and indicine breeds, to identify candidate selective sweeps in these populations, and assess their enrichment for distinct functional elements.

## Methods

### Samples

We retrieved 440 whole-genome sequences from the 1000 Bull Genomes Project (Run6, *Bos taurus,* and *Bos indicus*) for the 18 breeds that were chosen to constitute the reference population for imputation (Table 1) [24, 25]. The dataset contained 186 European taurine, 102 Asiatic indicine and 80 crossbred genomes as well as a subset of African samples from 12 taurine, 41 Sanga (ancient stabilized taurine × indicine crossbred [26], and 19 indicine individuals (Table 1). These breeds were selected to capture the lineages that are relevant to the beef industry since most tropical beef cattle are a genomic mosaic of indicine, African Sanga, European and African taurine cattle [27–29]. Thus, no dairy breeds were included in the study. Breeds were grouped according to their phenotypes and to known genomic crosses, i.e. taurine (humpless), indicine (with hump), admixed or African Sanga, the two latter being stabilized composite breeds [27, 30–33]. The selected animals were sequenced on an Illumina HISeq sequencer at an average coverage of 11.68 that ranged from 1.84 to 44.17.

**Table 1:** Whole-genome sequences used in the study

Name	Animal source	Sample size	Genome of origin
Brahman	Australia	90	*B. indicus*
Nelore	Brazil	5	*B. indicus*
Gir	Brazil	6	*B. indicus*
Shahiwal	India/Pakistan	1	*B. indicus*
Composite	Australia	56	Indicine-taurine
Brangus	USA	5	Indicine-taurine
Santa Gertrudis	USA	4	Indicine-taurine
BeefMaster	USA	15	Indicine-taurine
Charolais	France	128	*B. taurus*
Angus	Great Britain	51	*B. taurus*
Shorthorn	Great Britain	5	*B. taurus*
British shorthorn	Great Britain	2	*B. taurus*
N’ Dama	Africa	12	*B. taurus*
Uganda-mix	Africa	26	Sanga
Africander	Africa	5	Sanga
Ankole	Africa	10	Sanga
Ogaden	Africa	9	*B. indicus*
Boran	Africa	10	*B. indicus*

### Mapping, variant detection and imputation

The selected genome sequences were processed through the 1000 Bull Genomes Project pipeline [34]. Before sequence alignment, data were trimmed for adaptor sequences using Trimmomatic [35] and reads with a Phred quality score lower than 20, or with a read length shorter than 50% of the standard length were discarded.

The genome sequences were aligned to the UMD3.1 reference genome [36] with the BWA-MEM algorithm, using default parameters [37]. Duplicates were removed using Picard’s MarkDuplicates tools (http://broadinstitute.github.io/picard/) and local realignment of the reads around InDels was done with the GATK [38] tool IndelRealigner. Variant calling was performed by applying the GATK tool Best Practises [38]. All raw variants were called with the GATK [38] tool HaplotypeCaller based on the *Bos taurus* reference genome UMD3.1 and all raw variant VCF files were combined via the Genotype GVCF tools to produce a single VCF file. Genetic variants from the sequenced animals were extracted and filtered to retain only bi-allelic variants that had at least four copies of the minor allele. Sequences of the filtered variants were phased and imputed with the Eagle [39] and FImpute 2.2 [40] software, respectively. The analysis resulted in the detection of 39,679,303 high-quality SNPs, of which 24,080,747 were considered common SNPs (minor allele frequency (MAF) ≥ 0.05). Genetic diversity estimates were obtained by using PLINK v1.9 and PCA (https://www.cog-genomics.org/plink2) [41]. The VCFtool v.0.16 (–het) was used to calculate the observed homozygosity and heterozygosity as well as the inbreeding coefficient, F, for each individual [42]. Individual heterozygosity and F-values were plotted per breed and genome of origin using R version 3.5.2.

### Selective sweeps

Allele frequency differences between taurine and indicine populations were measured using the F_ST_ index (Weir and Cockerham method [43]). Average F_ST_ values were plotted in 20-kb overlapping genomic bins (with a number of SNPs > 10) with a 10-kb step-size. Nucleotide diversity (π) was measured in each population within the same 20-kb genomic bins. The ratio of indicine to taurine π was used to identify differences in nucleotide divergence between populations. The combined analysis of F_ST_ and π ratio (indicine/taurine) was used to identify candidate sweeps. The Z-transformed product of F_ST_ and π ratio values was declared significant if the genome-wide threshold was higher than 5.08, which represents a Bonferroni adjusted p-value lower than 0.05.

### Biological and phenotypical enrichment analysis

We performed a locus-based gene ontology enrichment with the GREAT v.3.0.0 software package [44]. Candidate selective sweeps (bins and/or regions) were translated to human coordinates (GRC37/hg19) using the liftOver tool (minMatch = 0.1) [45]. GREAT associates regions to genes and then performs a binomial (gene) and a hypergeometric test (region) to calculate the enrichment for biological terms, processes, Mouse Genome Informatics (MGI) database phenotypes, and Human Phenotype Ontology from OMIM. The default option ‘Basal plus extension’ association rule assigns genomic regions with genes, i.e. each gene is associated to a basal regulatory domain that extends 5 kb upstream and 1 kb downstream of the transcription start site (TSS) (regardless of the other nearby genes). In addition, each gene has an extended regulatory domain in both directions up to 1000 kb or until the basal domain of the nearest gene.

### Functional annotation of the cattle genome

We used the UMD3.1. version 1.87 assembly of the bovine genome and derived the following functional annotation tracks:

- Gene: gene coordinates expanding the exonic and intronic regions of a gene.
- CDS: coding sequences coordinates within a protein-coding gene.
- Intron: intronic coordinates were calculated as gene coordinates minus the CDS regions.
- Intergenic regions: whole-genome regions absent of gene coordinates annotation.
- 1-kb upstream: 1-kb regions upstream of the transcription start site (TSS) of the annotated protein-coding gene.
- 1-kb downstream: 1-kb regions downstream of the transcription end site (TES) of the annotated protein-coding gene.
- UTR: 3′ and 5′ UTR regions.

Next, we used predicted regulatory elements from our previous study [20] in which human regulatory elements from three distinct human regulatory databases, i.e. ENCODE, FANTOM and Epigenomics Roadmap, were projected onto cattle coordinates by reciprocal liftOver (minMatch = 0.1) [45]. The original or full set was further processed by applying different filters and thresholds including those for expression in bovine tissues [20]. The following datasets were included in the study:

- Human Projection All dataset: all predicted regulatory elements, proximal (promoters) and distal regulatory elements projected onto the bovine genome from three human databases, ENCODE, FANTOM, Epigenomics Roadmap. No filtering.
- Human Projection Proximal Elements: all proximal (promoter) regulatory elements from the same three databases.
- Promoter: FANTOM5 promoter atlas that was generated experimentally with CAGE data from almost 1000 tissues and cell lines [46] and projected onto cattle coordinates [20]. CAGE is a methodology for the detection of core promoter regions that bind the transcriptional machinery [47].
- Human Projection EnhG: all genic enhancers (EnhG) regions from the Epigenomics Roadmap database [23]. EnhG are enriched for H3K4me1 and H3K36me3 chromatin marks and correspond to enhancers that overlap with exonic regions [23].
- Human Projection EnhBiv: enhancer bivalent (EnhBiv) regions from the Epigenomics RoadMap database [23]. EnhBiv are associated with H3K4me1 and H3K27me3 chromatin marks [23].
- Human Projection Enh: enhancers (Enh) regions that are detected in the RoadMap Epigenomics database. Such enhancers are associated with H3K4me1 chromatin marks and tend to be distal regulatory elements [23].
- Human Projection Proximal transcription factor binding sites (TFBS): proximal TFBS from the ENCODE dataset [21].
- Human Projection Distal TFBS: distal TFBS from the ENCODE dataset [21].
- Human Projection Filtered set: whole dataset projected onto cattle coordinates after filtering.

Finally, we exploited publicly available experimental epigenomic marks present in the cattle genome, including:

- ATAC-seq cattle FR-AgENCODE data: the assay for transposase accessible chromatin (ATAC) identifies nucleosome-depleted regions in the genome, which are enriched for regulatory functions. The FR-AgENCODE pilot study performed ATAC-seq in CD4+ and CD8+ cells (http://www.fragencode.org/results.html) [48].
- Experimental chromatin marks in the liver obtained from a comparative analysis across 20 mammalian species [49], i.e. the ArrayExpress database with accession number E-MTAB-2633).
- Cattle H3K4me3: genomic coordinates that are significantly enriched for H3K4me3 chromatin marks in the *Bos taurus* liver. H3K4me3 is associated with promoter regions.
- Cattle H3K27ac: genomic coordinates that are significantly enriched for H3K27ac chromatin marks in the *Bos taurus* liver. H3K27ac is associated with active regulatory function.
- Cattle H3K27ac only: genomic regions that are significantly enriched for H3k27ac chromatin marks but with no enrichment for H3K4me3 chromatin marks in the *Bos taurus liver.*

### Assessment of the functional enrichment of selective sweeps

To assess the enrichment of genomic region sets, i.e. selection sweeps, for various functionally annotated genomic elements within the cattle genome, we used the R/Bioconductor package locus overlap analysis (LOLA) [50]. This tool requires (i) a ‘query set’, which is the list of genomic regions to be tested for enrichment.; (ii) a ‘reference set’ or a list of genomic regions to be tested for overlap with the ‘query set’; and (iii) a ‘universe set’, which is a background set of regions that could have been included in the query set. LOLA performs a Fisher’s exact test with a false discovery rate correction to assess the significance of the overlap in each pairwise comparison between the ‘query set’ and each entry in the ‘reference set’ [49]. We investigated the enrichment of detected candidate sweeps (query set) for a collection of distinct cattle functional elements (reference set). These include annotations (i) that are derived from the reference assembly UMD3.1 v.1.87; (ii) on predicted regulatory elements in the cattle genome based on the translation of human epigenomic marks coordinates from ENCODE and RoadMap epigenomics [20]; and (iii) on experimentally available epigenetic marks for the cattle genome [49] and Fr-AgENCODE ATAC-seq datasets [48]. The universal set was defined as a list of 20-kb genome-wide bins that were inputted in the selection sweep analysis and could potentially be found under selection.

### Analysis of divergent allele frequencies between populations and across functional categories

To assess whether divergent SNPs between populations were enriched for certain functional categories, we estimated the reference allele frequency (RAF) per SNP and per population using VCFtools (–freq) [42]. Then, we calculated their absolute allele frequency difference between populations ΔAF = abs (AF_taurine_ − AF_indicine_). Next, we binned SNPs by ΔAF in steps of 0.1 i.e. (ΔAF = 0.00–0.10, 0.01–0.20, etc. up to 0.90–1.00) resulting in 10 bins. These bins were intersected with functional categories i.e. coding exons, intronic, intergenic regions, etc., as described in the Cattle functional annotation methods section. For the ΔAF bins, the proportions of SNPs in each functional category were determined by using the software bedtools intersect [51]. M-values (log2-fold change) of the relative frequencies of SNPs in each functional category were calculated by comparing the frequency of SNPs per functional category in a specific bin with the corresponding frequency across all bins (expected value). Statistical significance of the deviation from the expected values was assessed using a Chi squared test. It should be noted that the F_ST_ at the SNP level, which is a measure of population divergence that accounts for ΔAF across populations, and the variance of the allele frequency within each population, could have been used for the analysis and binning. In our study, since both metrics were highly correlated (r^2^ = 0.975, we chose ΔAF, which is easier to use [17, 52]).

### *HELB* allele frequency across 2707 animals from the 1000 Bull Genomes project

Data processing and variant calling for the 1000 Bull Genomes sequences are described in [25]. Run6 of the project (released on March 2017) included 2707 animals from 97 breed groups, with 2379 animals classified as *taurus* and the remainder as unknown, *indicus*, or admixed [25]. We calculated allele frequencies per breed based on the breed classification provided by the 1000 Bull Genomes project.

### *HMGA2*-CNVR

We exploited a collection of in-house whole-genome sequence data from commercial breeding animals including five Africander, 56 tropical composites and 10 Brahman. All these animals are part of the 1000 Bull Genomes Project data collection. DNA was extracted from either blood or semen samples from each animal following a standard protocol. Paired-end short insert libraries were sequenced on the Illumina HiSeq 2000 platform. Reads were mapped against the cattle reference assembly UMD3.1/bosTau6 [36] using the BWA aligner v0.7.1 (bwa mem, default parameters) [37]. Duplicates reads were marked using Picard tools (http://broadinstitute.github.io/picard/). We assessed the existence of copy number variants (CNV) in the known *HMGA2*-CNV region on chromosome 5 between 48,074,233 and 48,080,443 bp (~ 6.2 kb) [53] by comparing the coverage in the CNVR versus the coverage along the whole chromosome 5. In addition, the alignments of all 71 animals were visualized with Integrative Genomics Viewer (IGV) [54] to confirm the existence of reads that harbor a duplication of the HMGA2-CNV.

## Results

### Genetic variation between taurine, indicine and admixed cattle

**Fig. 1:**
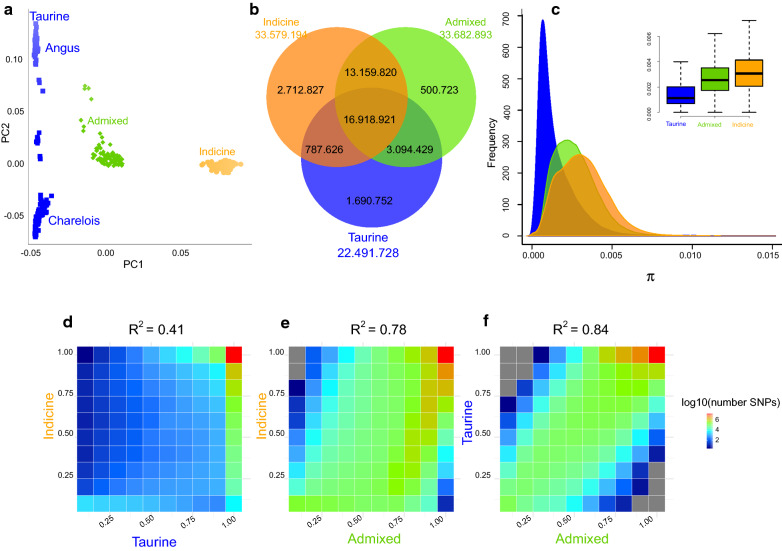
Genetic variation and divergence. **a** PCA of the genetic distance was performed to assess the clustering of sequences according to their genome of origin, taurine (blue), indicine (orange) or admixed (green). For PCA breed composition, (see Additional file 1: Figure S2). **b** Proportion and number of private and shared SNPs for the taurine (blue), indicine (orange) or admixed (green) cattle datasets. **c** Estimated nucleotide diversity in 20-kb genomic bins for the taurine (blue), indicine (orange) or admixed (green) sequences. **d**–**f** Correlations between estimated reference allele frequencies (RAF) between taurine (blue) and indicine (orange) (**d**), admixed (green) and indicine (orange) (**e**) and admixed (green) and taurine (blue) (**f**). An increasing number of SNP counts is related to warmer colours

To evaluate the genomic relationships between samples, we performed a principal component analysis (PCA) across all samples and datasets (see Additional file 1: Figure S1). In agreement with previous reports [55–57], PC1 (84.02% of variability) captured the taurine/indicine origin and PC2 (11.60% of variability) captured the African origin of the samples. The same PCA without the African samples resulted in PC1 capturing the taurine/indicine origin (77.74% of the variability) and PC2 (7.92% of the variability) dividing the taurine breeds along the Angus Charolais axis (Fig. 1a) and (see Additional file 1: Figure S2). Since the African samples represented a much smaller dataset (Table 1), we report the comparison of the African taurine versus indicine cattle, separately.

European and Asiatic breed variant calling resulted in 38,865,098 high-quality SNPs, of which 16,918,921 were shared among the three indicine, admixed and taurine populations (Fig. 1b). The number of private SNPs was larger for the indicine breeds (2,712,827) than for the taurine (1,690,752) or the admixed (500,723) breeds (Fig. 1b). The nucleotide diversity (π) and heterozygosity (het) values were higher for the indicine breeds (π = 0.32% and het = 0.20) than for the admixed and taurine breeds (π = 0.27% and 0.15% and het = 0.18 and 0.09, respectively) as shown in Fig. 1c and Additional file 1: Figure S3. The coefficient of inbreeding F was lower for indicine (− 0.07) and admixed (0.05) than for the taurine breeds (0.50), as shown in Additional file 1: Figure S4. The same trend was observed for the samples from Africa (indicine cattle: 1,767,058 private SNPs, π = 0.32%, het = 0.21, F = − 0.11; Sanga cattle: 3,794,711 private SNPs; π = 0.29%, het = 0.19, F = 0.006; taurine N’Dama: 510.325 private SNPs; π = 0.17%, het = 0.12, F = 0.34; (see Additional file 1: Figures S3–S5). We observed a smaller number of private SNPs and a lower nucleotide diversity for the taurine than the indicine breeds, which agrees with the more intense artificial selection of their production systems and with the evidence of taurine introgression in indicine cattle [56, 58, 59].

Finally, we observed that although most SNPs were common between indicine and taurine breeds, the correlation of the reference allele frequency (RAF) bins between these two breeds was low (R^2^ = 0.41), compared to that between indicine and admixed (R^2^ = 0.78) and between taurine and admixed (R^2^ = 0.84) breeds (Fig. 1d–f). Similar results were found for African cattle (see Additional file 1: Figure S5). This indicates that the genomic divergence between indicine and taurine breeds is higher than between domestic sheep (*Ovis aries*) and their wild counterpart (Mouflon *Ovis orientalis*) (R^2^ = 0.79) [18].

### Genomic regions under selection in European taurine and Asian indicine cattle

**Fig. 2:**
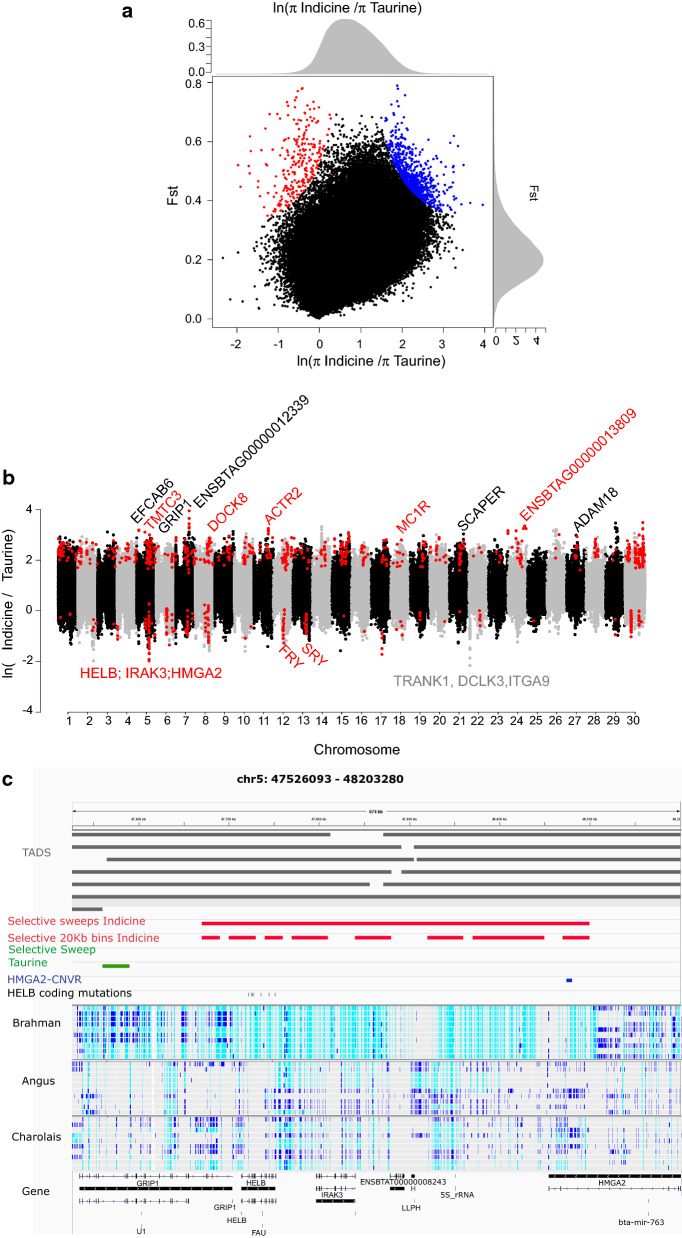
Candidate selective sweeps in taurine and indicine cattle. **a** Population differentiation index (F_ST_) and relative nucleotide diversity between taurine and indicine cattle in genome-wide 20-kb genomic bins. Outlier bins that show evidence of selection in taurine breeds (blue) and indicine breeds (red). **b** Genome-wide distribution of relative nucleotide diversity. Positive and negative values represent candidate sweeps in taurine and indicine cattle, respectively. Outliner bins are coloured in red. **c** IGV screenshot of chr5: 47,526,093–48,203,280. In red, the 430 kb long selective sweep in Asian indicine cattle: spanning *GRIP1*, *HELB*, *IRAK3*, *ENSBTAG00000026993*, *LLPH*, and part of *HMGA2*. In green, a selective sweep in *GRIP1* in European taurine cattle. In blue, the 6.2 kb tandem duplication *HMGA2*-CNVR reported by [53]. Below 10 variant files in vcf format for 10 Brahman animals, 10 Angus, and 10 Charolais

By pooling genomes into groups of subspecies and comparing the patterns of variability, we sought to identify genomic regions and genes that are putatively involved in their phenotypic and behavioural differences. The F_ST_ (see Additional file 1: Figure S6) and the ratio of indicine to taurine π were plotted for each 20-kb genomic bin (Fig. 2a, b) and (see Additional file 2: Table S1), revealing 657 candidate bins under selection in the taurine genome and 242 in the indicine genome (P-adj < 0.05) (see Additional file 3: Table S2 and Additional file 4: Table S3). Bins that were closer than 50 kb apart were merged, which yielded 376 and 72 candidate selective sweep regions, for the taurine and indicine genome, respectively (average sizes of 29,5 kb, and 52,5 kb, respectively) (see Additional file 5: Table S4 and Additional file 6: Table S5).

Inspection of the gene content in the taurine selective sweep regions revealed several genes that are known in cattle or other species. For example, for the *melanocortin 1 receptor* (*MC1R*) gene, we found low π and high F_ST_ in taurine cattle, which is consistent with values reported in the literature for taurine cattle, horses and pigs in studies on coat pigmentation patterns [8, 60, 61]. The *leucine*-*rich repeats and immunoglobulin*-*like domains protein 3* (*LRIG3*) gene is known to be under selection in Charolais cattle (a predominant taurine breed in our study) and has been associated with elongated body axis [8]. Another gene of interest is the *myosin 1A* (*MYO1A*) gene that is known to be under divergent selection between taurine and indicine breeds and to influence pigmentation [61, 62].

Few outlier regions and genes were detected in the genomes of indicine cattle, but within those regions, our results confirmed several previously reported genes, such as *LEM domain*-*containing protein 3* (*LEMD3*) on *Bos taurus* (BTA) chromosome 5 (BTA5) [8]. A major finding was a large selective sweep that spans 430 kb on BTA5 (47,670,001–48,100,000 bp) (Fig. 2c). This is the largest region under selection, which also displays the largest difference in π between indicine and taurine cattle (Fig. 2b, c) and (see Additional file 6: Table S5). This region is near fixation in indicine cattle and spans several genes including *HELB*, *IRAK3*, *ENSBTAG00000026993*, *GRIP1,* and part of *HMGA2* (Fig. 2c). Published genome-wide association studies (GWAS) in tropical cattle, associated this region with traits including sheath score and yearling weight [63] or reproductive traits in tropical cattle [64–66]. Finally, within this candidate indicine selective sweep, a tandem duplication of ~ 6.2 kb (48,074,233–48,080,443 bp) was reported to affect the third and fourth introns of *HMGA2* in Nellore cattle and to be associated with navel length (similar to sheath score) at yearling (Fig. 2c) [53]. Taken together, these results indicate this 430-kb selective sweep is relevant for selective breeding programs aimed at improving adaptation of cattle to tropical conditions.

### Genomic regions under selection in African cattle

Analysis of African whole-genome sequences (see Additional file 1: Figure S7 and Additional file 7: Table S6) resulted in the detection of 1194 20-kb bins for African taurine cattle (N’dama n = 12) and 324 in African *Bos indicus* (Boran n = 10, Ogaden n = 10) (see Additional file 8: Table S7 and Additional file 9: Table S8. After merging the 20-kb bins less than 50 kb apart, we defined 611 and 117 genome-wide regions under selection in African taurine and indicine breeds, with an average size of 35.5 and 42.1 kb, respectively) (see Additional file 10: Table S9 and Additional file 11: Table S10).

African taurine cattle (N’Dama) inhabit regions that are infested with tsetse fly, and thus, have evolved mechanisms to tolerate trypanosoma infection, including resistance to anaemia, its major clinical sign [67]. Our analysis captured genes that are associated with resistance to anaemia, under selection in African taurine cattle, and potentially related to trypanotolerance in cattle (see Additional file 10: Table S9). These include, *erytrocyte membrane protein brain 4.1* (*EPB41*), which encodes proteins of the red cell membrane skeleton and is associated with hematologic disorders in humans related with variable degrees of anaemia [68], and *ferroportin* (*SLC40A1*), a gene that is relevant for iron homeostasis [69] and was previously reported to be under selection in African taurine cattle [14].

### Biological processes and phenotypes associated with candidate selective sweeps

Analysis of the taurine populations, revealed only two significantly enriched biological processes terms: regulation of catenin import to the nucleus (binomial test FDR q-value 1.46 × 10^−3^, hypergeometric test FDR q-value 1.92 × 10^−2^) and embryonic skeletal joint development (Binomial test FDR q-value 3.38 × 10^−2^, hypergeometric FDR q-value 3.65 × 10^−2^). At the phenotype level, we found a significant enrichment of candidate selective sweeps for mouse behavioural traits (Table 2) and (see Additional file 12: Table S11) and the top term was “increased exploration in new environment” [binomial test p-value 2.08 × 10^−14^, Table 2 and (see Additional file 12: Table S11)], which is consistent with the reported behavioural differences between taurine and indicine cattle [70–74]. The other enriched terms for mouse phenotypes in regions under selection in taurine cattle relate to changes in pigmentation such as belly spot or hypopigmentation (see Additional file 12: Table S11). These results are consistent with the objectives of artificial selection for colour patterns in many species including cattle [75], pig [76, 77], horse [78, 79], and sheep [80]. In contrast, the only enrichment associated with indicine candidate sweeps was for human phenotypes related to body height (binomial test FDR q-value = 8.9 × 10^−17^, hypergeometric test FDR q-value = 2.91 × 10^−02^, GREAT v 1.8 Human Phenotypes), which involves genes such as *HMGA2*, *KDM6A*, *LEMD3*, *FERMT1* (see Additional file 13: Table S12). In cattle, body weight is a trait that has been subject to various selection pressures over time and across breeds [81–83].

**Table 2:** Top 10 enriched terms from Mouse Genome Informatics (MGI) phenotype in GREAT for the identified *Bos taurus* selective sweeps in the comparison *Bos indicus versus Bos taurus*

Term name	Binomial raw p-value	Binomial test FDR Q-value	Binomial fold enrichment	Binomial observed region hits	Binomial region set coverage	Genes
Increased exploration in new environment	2.08 × 10^−14^	1.65 × 10^−10^	7.11	26	0.04	*DRD3*, *FMR1*, *GRIA2*, *NPAS3*, *OPHN1*, *SH3KBP1*
Decreased aggression	5.73 × 10^−11^	1.13 × 10^−07^	5.40	24	0.04	*ARX*, *ESR1*, *FMR1*, *GRIA2*, *MAP6*, *NDUFS4*, *OPHN1*
Abnormal kidney interstitium morphology	1.25 × 10^−08^	1.10 × 10^−05^	4.87	20	0.03	*AGTR1*, *COL4A3*, *KIF3A*, *NPHP3*, *PDGFRA*, *TNFRSF1B*, *TRPS1*, *XDH*
Abnormal social investigation	9.88 × 10^−07^	2.17 × 10^−04^	3.42	22	0.03	*AVPR1A*, *EXT1*, *FMR1*, *GRIA4*, *LRRTM1*, *MAGED1*, *MAP6*, *NBEA*, *NPAS3*
Abnormal strial marginal cell morphology	1.64 × 10^−06^	3.09 × 10^−04^	10.25	8	0.01	*COL4A3*, *ESRRB*, *KIT*, *NDP*, *SLC12A2*
Abnormal startle reflex	1.87 × 10^−06^	3.44 × 10^−04^	2.37	37	0.06	*BRE*, *CTNNA2*, *DRD3*, *ESRRB*, *FMR1*, *GLRB*, *GPR98*, *GRIA4*, *MECOM*, *MRO*, *NDUFS4*, *NPAS3*, *PHYKPL*, *SLC12A2*, *SLITRK6*, *TNFRSF1B*
Abnormal frontal bone morphology	8.19 × 10^−06^	9.55 × 10^−04^	2.89	23	0.04	*BMP4*, *DISP1*, *EFNB1*, *HDAC8*, *HHAT*, *KIF3A*, *MSTN*, *NOG*, *PDGFRA*, *SATB2*, *SP3*, *WNT9A*
Abnormal lens induction	4.21 × 10^−05^	3.00 × 10^−03^	4.20	12	0.02	*BMP4*, *GRIP1*, *MAB21L1*, *PAX6*, *SOX1*
Abnormal pain threshold	5.29 × 10^−05^	3.57 × 10^−03^	2.03	37	0.06	*ADAMTS5*, *AFF2*, *ARX*, *BAMBI*, *EDNRB*, *ESR1*, *EXT1*, *FMR1*, *GABRR1*, *GNAQ*, *GRIA2*, *GRIA4*, *HTR1F*, *LMO7*, *MC1R*, *NDUFS4*, *OPRK1*, *TRPM3*
Abnormal fear-related response	8.43 × 10^−05^	5.09 × 10^−03^	2.98	17	0.03	*ARX*, *ESR1*, *EXT1*, *FMR1*, *GRIK2*, *MAP2*, *SLITRK1*

242 20-kb windows p- value < 0.05

No functional significant term was enriched in African cattle selective sweeps as previously reported [14].

### Functional annotation associated with candidate selective sweeps

**Fig. 3:**
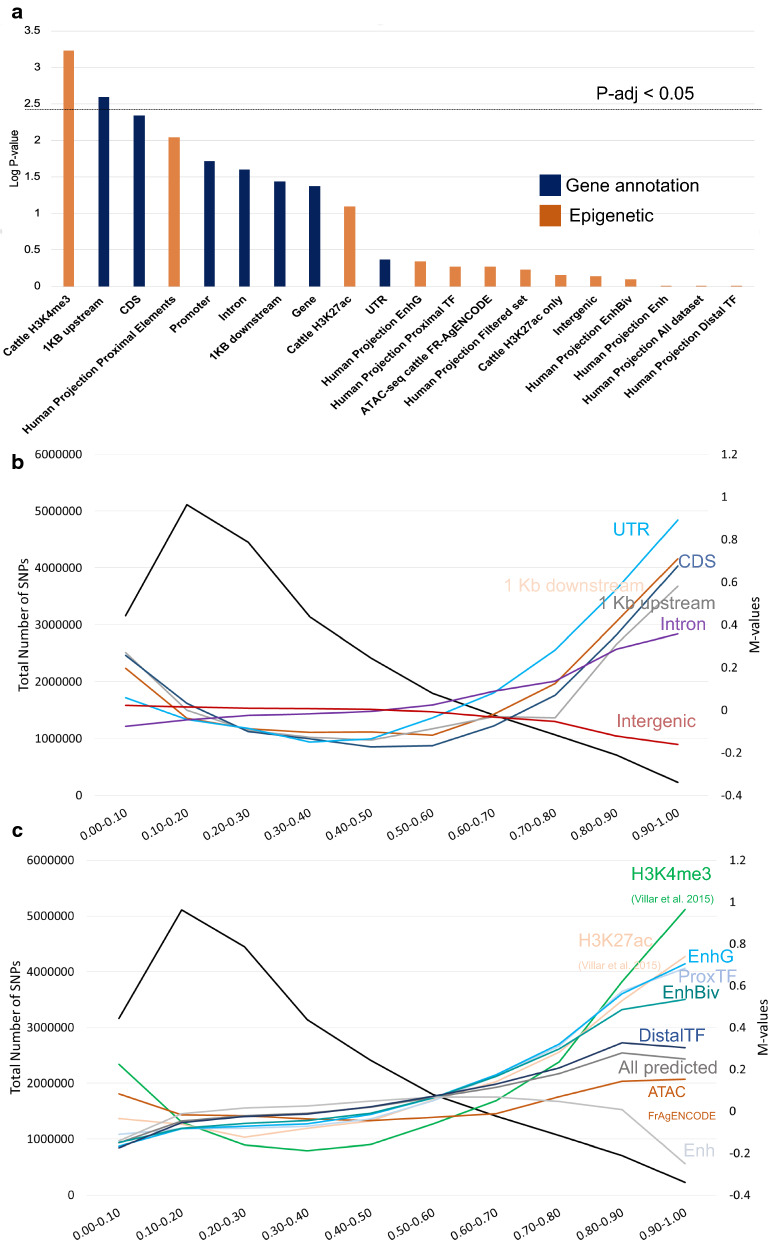
Genomic feature enrichment in selective sweeps. **a** The strength of enrichment for 20 genomic features in 72 indicine-specific regions assessed by overlapping genomic regions [50]. **b** Intersection of the delta allele frequency (ΔAF) with functional annotations derived from the reference UMD3.1 bovine genome. The number of SNPs in ΔAF bins is indicated on the left, and the *M*-value (log2-fold change) of the relative frequencies of SNPs in each functional category (on the right). The black line shows the number of SNPs within each (ΔAF) bin. **c** Intersection of the delta allele frequency (ΔAF) with functional annotations from the predicted regulatory elements in the cattle genome [20] and publicly available experimental epigenetic marks [49] and Fr-AgENCODE [48]

Selection can differ depending on distinct genomic functional elements, such as coding elements or regulatory elements, which are mostly related to changes in gene expression. To tackle this issue, we investigated the enrichment of previously detected candidate sweeps for a collection of experimental and predicted cattle functional elements (Fig. 3a) and (see Additional file 1: Figure S8, Additional file 14: Table S13 and Additional file 15: Table S14). No functional enrichment was observed in taurine candidate sweeps (n = 357) (see Additional file 1: Figure S8 and Additional file 14: Table S13). However, regions under selection in the indicine cattle (n = 72) presented a significant enrichment for proximal and genic features (Fig. 3a) and (Additional file 15: Table S14). This is indicated by the enrichment for experimentally defined promoters that were identified by H3K4me3 analysis in bovine liver tissue [49], and for 1-kb upstream genic and intronic regions from the current UMD3.1 v.187 (Fig. 3a) and (Additional file 15: Table S14). Analysis of African cattle selective sweeps also showed a significant enrichment for proximal features including 1-kb upstream UTR and EnhG, which are regions reported as enhancers but are overlapping gene bodies [23] (Fig. 3a) and (Additional file 16: Tables S15 and Additional file 17: Table S16). Taken together our results agree with the findings of previous studies on sheep domestication, which concluded that the major differences between domestic and wild sheep genomes concern functional elements close to genes rather than intergenic or distal enhancers [18].

### Site frequency analysis

To complement our scan for selective sweeps in 20-kb bins and to exploit all the information from whole-genome sequences, we studied the differences in allele frequencies between European taurine and Asian indicine populations for 23,494,872 SNPs and between African taurine and indicine for 22,943,179 SNPs (Fig. 3a) and (Additional file 18: Tables S17 and Additional file 19: Table S18). We found that only a small proportion of each set of SNPs, i.e. 228,908 (0.97%) and 26,561 (0.11%) SNPs, respectively, presented a ΔAF higher than 0.9, which indicates that they are close to fixation between the European taurine and Asian indicine populations, and between the African taurine and indicine populations, respectively. Given the high level of divergence and the comparatively low correlation of allele frequencies between taurine and indicine cattle (Fig. 1) and (Fig. 3a) and (see Additional file 1: Figure S5), sorting the sites under selection from those that display high ΔAF due to drift, is a challenging issue. Functional enrichment analysis (Additional file 19: Table S18) confirmed our previous analysis at the level of genomic bins (Fig. 3a), since we observed a clear enrichment for UTR, coding regions and proximal regions such as promoter regions identified by H3K4me3 analysis in cattle liver [49], and predicted enhancer genic regions (Fig. 3b, c). The same analysis in African cattle (Additional file 1: Figure S9, Additional file 20: Table S19 and Additional file 21: Table S20) agreed with these results.

### Fixed coding mutations in the *HELB* gene in indicine cattle

**Fig. 4:**
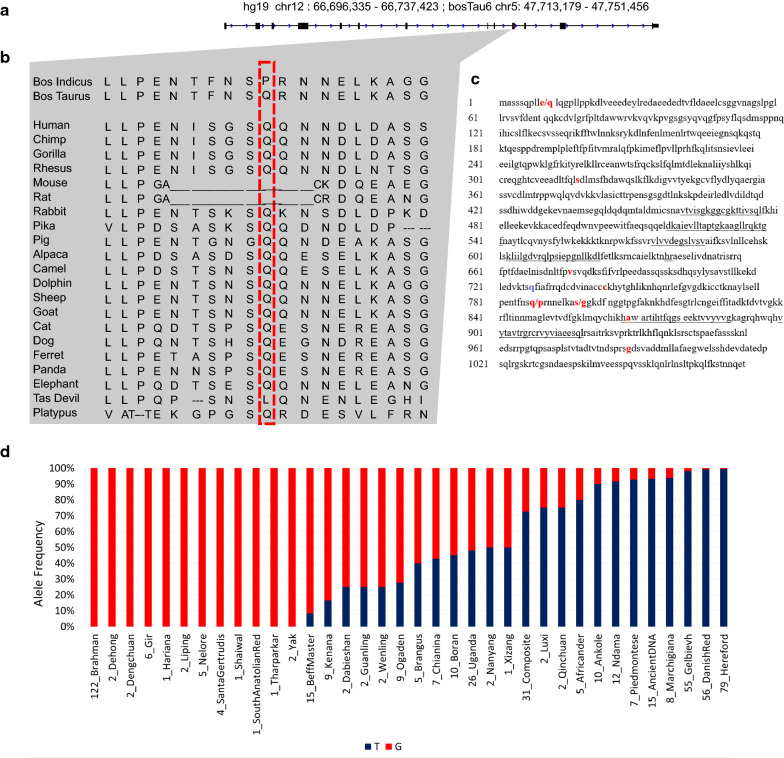
Candidate SNPs in the *HELB* gene. **a** Representation of the *HELB* gene based on human hg19 coordinates (chr12: 66,696,335–66,737,423) and bovine bosTau6 coordinates (chr5:47,713,179–47,751,456). **b** Multiple sequence alignment of amino acids across representative mammalian species. In red: the candidate indicine-specific mutations. **c** HELB amino acid sequence with the indicine-specific mutations are highlighted in red. Non-synonymous mutations with substitution residues are located at positions 10, 788 and 791. In purple, residue 428, which had been previously associated to temperature sensitive murine cell lines [88]. **d** Allele frequency of the SNP rs447470311 located in chr5:47726121 across the 36 breeds in Run6 1000 Bull Genomes Project which contain the G variant [24]

Comparison of the European taurine and Asian indicine genomes showed that a small proportion of the variants assessed (926 loci or 0.004% of those tested) were fixed for different alleles (ΔAF = 1). Annotation of these 926 loci revealed that only nine of them were located in exons (Additional file 22: Table S21). We detected one synonymous mutation on chromosome X at 143,768,373 bp in *ENSBTAG00000048102* or *OFD1Y* and eight mutations, three missense and five non-synonymous that were located within the *HELB* gene (Fig. 4a–c) and (see Additional file 18: Table S17) on BTA5 (47,713,856–47,751,469 bp), which is within the previously reported 430-kb selective sweep on this chromosome (47,670,001–48,100,000) (Fig. 2c). HELB functions as an ATP-dependent DNA helicase that is involved in DNA damage response [84, 85] and facilitates the recovery of the cells from replication stress during the S phase [86]. Non-synonymous mutations in *HELB* have been associated with male and female reproductive traits in tropical cattle [66] and with Xeroderma pigmentosum, complementation group B, a skin pigmentation disorder in humans leading to solar hypersensitivity of the skin [87]. In addition, point mutations in the *HELB* coding sequence have been identified in murine cell lines with temperature-sensitive DNA replication (Fig. 4c) [88]. Taken together, the mutations in *HELB* could lead to a modification of its DNA damage response function to better cope with different cell stresses associated with indicine tropical environments such as constant high temperatures and high levels of UV intensity.

In African cattle, only four fixed coding mutations were identified between taurine and indicine populations (see Additional file 19: Table S18): one missense mutation in the *NR4A1* gene (BTA5:27,982,214), which encodes a fibroblast growth factor involved in ovarian function [89], and three synonymous mutations in the coding regions of *ENSBTAT00000005937*, *DRP2* and *ADCK2* (Additional file 23: Table S22). Previously reported mutations in *HELB* were shown to be present in both taurine (N’ dama) and indicine African cattle (Additional file 19: Table S18).

### Confirmation that point mutations in *HELB* are specific to indicine cattle

To examine whether mutations in the *HELB* gene are indicine-specific in a wider collection of breeds, we estimated the allele frequency of the *HELB* coding variant with the highest SIFT effect, i.e. rs447470311 (BTA5:47,726,121) in all the individual whole-genome sequences retrieved from Run6 (March 2017) of the 1000 Bull Genomes Project [24], i.e. 2709 whole-genome sequences corresponding to 97 classified breed compositions [25]. We observed that only 36 breeds presented the *G* allele of rs447470311 corresponding to 100% of the indicine breeds or indicine admixed (Fig. 4d) and (see Additional file 1: Figure S10 and Additional file 24: Table S23). However, it should be noted that this allele was also found, at a lower frequency, in some European taurine breeds (Fig. 4d) and (see Additional file 1: Figure S10 and Additional file 24: Table S23), mostly Italian breeds, such as Marchiginana, Chinanina, Piedmontese, or Anatolian breeds, which are all known to have a history of indicine introgression [56, 58, 59].

Finally, by assessing samples of ancient DNA from the 1000 Bull Genomes Project (Run6), we found that allele *G* was present (with an allele frequency for *G* = 0.067) in 15 of the samples tested from animals dating back to the roman empire and medieval era [90, 91]. Based on this result, we inferred that the *G* allele has not persisted in the taurine lineage because either of genetic drift or negative selection within the European taurine breeds. This allele is also found in several current Iranian admixed individuals (allele frequency for *G *= 0.39, n = 9) and in Yak individuals (allele frequency for *G *= 1, n = 2) [25] (Fig. 4d) and (see Additional file 1: Figure S8 and Additional file 24: Table S23).

### Mutations in the *HELB* gene and the *HMGA2*-CNVR segregate independently

**Fig. 5:**
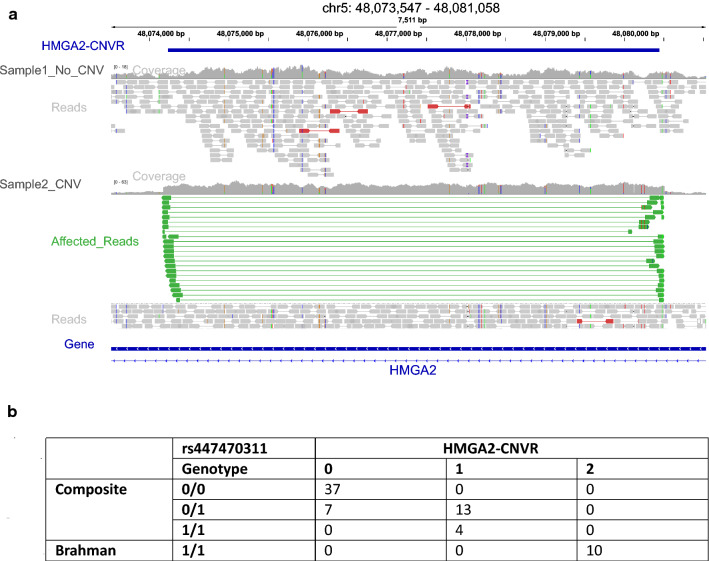
*HMGA2*-CNV. **a** IGV screenshot of the *HMGA2*-CNVR locus (in blue) and the visualization of the alignment of paired-end reads. Sample1_No_CNV shows the aligned reads of a composite animal presenting no CNV; Sample2_CNV shows the aligned reads of a Brahman animal presenting the duplication event. Paired-end reads in green imply duplication with respect to the reference genome. These read pairs are oriented towards the outer sides of the predicted insert. Red colour reads indicate paired end reads with an insert size larger than expected (possible deletion). **b** Contingency table of SNP rs447470311 (chr5:47,726,121) versus *HMGA2*-CNVR (chr5:48,074,233–48,080,443 (~ 6.2 kb) genotype

The *HELB* gene is located in a 430-kb selective sweep on chromosome 5 (47,670,001–48,100,000 bp) and is fixed in indicine cattle but not in taurine cattle (Fig. 2c). This region also includes *ENSBTAG00000026993*, *GRIP1* and part of *HMGA2* (Fig. 2b, c) and (see Additional file 1: Figure S11) for ARS-UCD1.2 coordinates). The latter gene is of particular interest because a 6.2-kb CNV that spans a segment of *HMGA2* intron 3 in Nellore (indicine) cattle is associated with navel score [53] (Fig. 2c). Thus, we investigated whether the entire selective sweep region, i.e. including *HELB* and *HMGA2*-CNVR, was in linkage disequilibrium or segregated independently, since independent segregation would explain the contribution of individual genomic elements in the region to multiple production traits in cattle. Towards this aim, we mapped bovine predicted topologically domains (TAD) in this region [92]. TAD are indicative of regions that physically interact more frequently with each other than with other sequences outside of the TAD [93]. We found that two predicted TAD were located in the 430-kb selective sweep: one spanning, *GRIP1*, *HELB*, *IRAK3* and *ENSBTAG00000026993*; and a second spanning *HMGA2* (Fig. 2c), which strongly suggests that *HELB* and *HMGA2* are located in two independent regulatory entities and segregate in an independent manner. Next, to confirm that the mutations in *HELB* and the *HMGA*-CNVR segregate independently, we genotyped by whole-genome sequencing 71 animals from commercial breeds including 10 Brahman cattle, 5 Africander and 56 tropical composite, and assessed their genotype for rs447470311 in the *HELB* gene and for *HMGA2*-CNVR (Fig. 5a). Our results show that Brahman cattle (100% indicine) are homozygous for the alternative allele (‘homozygous alternative’) of SNP rs447470311 and carry two copies of the *HMGA2*-CNVR (Fig. 5b) and (see Additional file 25: Table S24), whereas admixed or tropical composite animals displayed different combinations of genotypes at these two loci (Fig. 5b). Among the composite animals, all those that are homozygous for the reference allele at rs447470311, do not carry the *HMGA2*-CNVR. In contrast, all the animals that are homozygous for the alternative allele at rs447470311 carried one tandem repeat *HMGA2*-CNVR. It should be noted that, in our dataset, all Brahman cattle that were homologous at the rs447470311 alternative genotype carried two *HMGA2*-CNVR (Fig. 5b). Finally, animals that were heterozygous at rs447470311 carried either one *HMGA2*-CNV or no CNV. Thus, our results demonstrate that the genotype at the rs447470311 SNP in *HELB* and the *HMGA2*-CNVR segregate independently in admixed populations.

## Discussion

The marked phenotypic, physiological and behavioural differences between taurine and indicine cattle offer the opportunity to identify which genomic loci and genes shape these fundamental differences. In this study, our aim was to exploit the population history of the tropical beef cattle raised in Australia, which are a mixture of European taurine, Asian indicine, and animals from the African continent, to identify selection sweeps and identify the impact of selection across distinct functional categories.

At the level of selective sweeps, we identified genes that were previously reported under selection within European taurine breeds, such as *MC1R* or *MYO1A* that are involved in pigmentation [60–63]. *MYO1A* has also been identified to be involved in the growth hormone (GH) metabolism and in GH-related phenotypes such as body fat percentage in humans [94]. This is largely consistent with production-related traits in taurine cattle. In African cattle, we identified genes that are linked to the adaptation of N’dama to trypanosoma as shown in [14]. The two geographic comparisons described in this paper, reveal a larger number of regions under selection in taurine breeds than in indicine breeds, which suggests that selective pressures are stronger in taurine than in indicine cattle. This may reflect the consequence of the long-standing family-based breeding programs still underway in taurine breeds. We showed that orthologous selective sweep regions in European taurine cattle and mice are enriched for behavioural traits, mostly related to the exploration of the environment and fear response, which provides a better understanding of the impact of selection, (Table 2) and (see Additional file 12: Table S11). Thus, our results are consistent with the differences in temperament observed between taurine and indicine breeds [70, 71]. However, it should be noted that the specificity of the set of animals used in our study is likely to have an impact on the final collection of selective sweeps identified. For example, the European taurine selective sweeps were detected via a comparison to four indicine breeds (Brahman, Nellore, Gir and Sahiwal) that are mostly of Australian origin and raised under extensive systems with few human contacts. This animal set may represent patterns of variation that differ from those in a set of animals of the same four breeds but sourced from India, where populations are raised in small communities and with many human contacts. Such differences are observed when selective sweeps are explored in African taurine and indicine populations with no significant functional or phenotypic enrichment of sweeps.

A major evolutionary question concerns the relative contribution of coding or regulatory sequence evolution to the morphological and physiological divergence of species [95–97]. The lack of genomes with functional annotation has hampered the ability to address this question in livestock species. Our study is the first to measure the contribution of functional elements to the evolution of cattle. Our results conclude that selection and major differences in allele frequency between taurine and indicine cattle are driven by changes in proximal regulatory elements, promoters associated with H3K4me3 marks or located 1-kb upstream of protein-coding regions, which suggests that changes in gene expression have a major role in the divergence of taurine and indicine cattle. Our results are robust in terms of across-geography comparisons, i.e. European taurine against Asiatic indicine and African taurine against African indicine. In addition, they are in line with previous findings of studies on sheep domestication [18], and rabbit domestication in which an enrichment for conserved non-coding sites involved in regulatory functions and for coding regions has been shown [17]. Since distal regulatory elements tend to be tissue-specific and less evolutionarily conserved [22, 23, 49], our results on this type of elements could be an artifact due to an incomplete annotation of the bovine genome. We hope that, in the near future, the international efforts such as FAANG or of individual laboratories will advance the annotation of experimental functional elements, in particular those in distal positions, and help us to investigate more accurately the impact of distal tissue-specific and developmental regulatory elements.

At the coding level, we found only nine SNPs that were fixed in both European and Asiatic indicine breeds. None of these were nonsense or frame-shift mutations, which indicates that loss of function has not played a major role in the evolution of these two cattle subspecies, which is consistent with analyses in chicken [16], pigs [77], rabbit [17] and sheep [18]. Of these nine fixed SNPs, it is particularly remarkable that eight fall within coding regions of the *HELB* gene. An independent analysis pointed *HELB* as a relevant gene for adaptation of cattle to tropical conditions. In particular, several SNPs in this gene are associated with yearling weight in a tropical composite breed (BovineHD0500013787 on BTA5:47,724,746 explaining 5.35% of the genetic variance and with a − log(P) = 13.7) and with reproductive traits, scrotum circumference and puberty and post-partum anoestrus interval (BovineHD0500013788 on BTA5:47,727,773, explaining 2.50% and 3.87% of genetic variance, respectively, p-value < 10^−7^) [66, 67]. Furthermore, *HELB* is co-expressed with *MYO5A* [98, 99], which was recently shown to be associated with tick resistance in *Bos taurus*$\times$*Bos indicus* crossbred cattle [100]. We also showed that some SNPs are present only in the genome of cattle with indicine ancestry or introgression, including African cattle (indicine as well as taurine) and European ‘drought’ resistance breeds such as Red Anatolian, or Italian breeds, Marchigiana, Chianina and Piedmontese. Based on these findings, *HELB* is a likely major target for both human and natural selection for cattle to cope with a tropical environment.

The identification of causative genes and functional mutations is often complicated by linkage disequilibrium. Other mutations, including regulatory mutations in *HELB* that modify its expression pattern, or mutations in other genes such as *HMGA2*, could also affect tropical adaptation. Recently, a tandem repeat that includes the third and fourth introns of *HMGA2* has been associated with navel score and with visual scores of precocity and muscling in Nellore cattle [53]. We show that the mutations in *HELB* and the *HMGA2*-CNVR can be inherited independently, which means that they can potentially affect different phenotypes. Also, in previous studies, our group showed that the navel score trait associated with *HMGA2*-CNV has a low genetic correlation with yearling weight associated with *HELB*, in the composite and Brahman populations (R^2^ = 0.18 and 0.032, respectively) [64]. This provides additional support for the role of *HELB* in tropical adaptation.

Further studies are necessary to better assess the impact of coding mutations in *HELB*. One attempt to evaluate the phenotypic impact of such mutations is to exploit gene-editing technologies, such as CRISPR/Cas9 [101]. The editing of the *HELB* indicine specific mutation in taurine breeds could validate the beneficial role of the mutation to tropical adaptation. Finally, the identification of mutations in *HELB* will be useful to obtain more accurate genetic evaluations for prediction of crossbred cattle, a key challenge in the beef tropical cattle industry [102–104].

## Conclusions

We compared the genome sequences from European taurine and Asian indicine with those from African cattle and identified selective signatures between these cattle subspecies. We gathered publicly available experimental and predicted cattle functional annotation data and found that selective sweeps were enriched for promoter and coding regions. At the nucleotide level, sites that showed a strong divergence between taurine and indicine cattle were enriched for the same functional categories. In the genomes of the indicine cattle, we identified fixed SNPs that affect the coding sequence of *HELB*, which is located in a 430-kb selective sweep on chromosome 5. In addition, the *HELB* gene is involved in DNA damage response including exposure to ultra-violet light and thus, is relevant for tropical adaptation. Analysis of 2707 genomes from 97 breeds included in the 1000 Bull Genomes Project confirmed that *HELB* coding mutations were specific to indicine cattle. Finally, we showed that the mutations in *HELB* and the *HMGA2*-CNVR present in the same region segregated independently, which indicates that they can potentially affect distinct phenotypes.

## Supplementary information

**Additional file 1. Figure S1.** PCA of the genetic distance across all samples of European, Asian indicine, African taurine and African indicine cattle based on their genome (a) and (b) or breed of origin (c) and (d). (a) PC1 and PC2 explaining 84.02% and 11.60% of the variability, respectively; (b) PC1 versus PC2 (77.74% and 7.92% of the total variability for PC1 and PC2, respectively); and (c) and (d) PC1 versus PC2 and PC1 versus PC3 colour coded according to breed, respectively. **Figure S2.** PCA of the genetic distance to assess the clustering of sequences according to their breed of origin. (a) PC1 (77.74% of the total variability) and PC2 (7.92% of the total variability); and (b) PC1 versus PC3 (5.45% of total variability). **Figure S3.** Heterozygosity levels (number of heterozygous sites/total number of sites) in Asiatic, admixed and European taurine breeds classified according to genome of origin (i.e. orange = indicine, green = admixed, blue = taurine) (a) or to breed (b); and in African cattle classified according to genome of origin (c) or according to breed (d). **Figure S4.** Inbreeding coefficient, F, in Asiatic, admixed and European taurine breeds classified according to genome of origin (i.e. orange = indicine, green = admixed, blue = taurine) (a) or to breed (b); and in African cattle classified according to genome of origin (c) or to breed (d). **Figure S5.** Genetic variation and divergence in African cattle. (a) Proportion and number of private and shared SNPs in a set of African whole-genome sequences corresponding to 12 N’Dama (taurine in blue), 26 Uganda-mixed, 5 Africander and 10 Ankole (Sanga, zebu-taurine in green) and 10 Oganden and 10 Boran (Zebu or indicine in orange) (b) Nucleotide diversity was estimated in 20-kb genomic intervals for N’Dama π = 0.17%, Sanga π = 0.29% and indicine π = 0.32% sequences. Correlations between estimated reference allele frequencies (RAF) between taurine and indicine (c), Sanga and indicine (d) and Sanga and taurine (e). Bins were estimated for each genome of origin or population, then compared between populations and visualised in heatmaps. The colors get warmer as the number of SNP counts increases. **Figure S6.** F_ST_ measure in 20-kb genome-wide overlapping bins with a 10 kb step size. **Figure S7.** Candidate selective sweeps in taurine and indicine in African cattle. (a) Population differentiation (F_ST_) and relative nucleotide diversity between taurine and indicine cattle in genome-wide 20-kb genomic bins. (b) Genome-wide distribution of relative nucleotide diversity. Positive values represent candidate sweeps in taurine cattle and negative values in indicine. (c) F_ST_ measure in 20-kb genome-wide overlapping windows with a 10-kb step size. **Figure S8.** Genomic feature enrichment in selective sweeps. Strength of enrichment for 20 genomic features within 372 European taurine regions (a); 611 African indicine regions (b); 117 African taurine regions (c). **Figure S9.** Intersection of delta allele frequency (ΔAF) with different gene annotations. (a) Genome annotation derived and (b) using predicted [20] and experimental annotations in cattle [48, 49]. **Figure S10.** The 36 breeds in run6 of the 1000 Bull Genomes Project which present allele G at SNP rs447470311 (chr5:47726121) [24]. **Figure S11.** Regions that include selective sweeps in the cattle genome new assembly (ARS-UCD 1.2) at coordinates chr5: 47,481,051–47,520,235.

**Additional file 2: Table S1.** Taurine versus indicine F_ST_ and nucleotide diversity genome-wide in 20-kb overlapping windows with a 10 kb step size.

**Additional file 3: Table S2.** 657 detected selective sweeps (20-kb) bins in European taurine cattle compared to Asian indicine.

**Additional file 4: Table S3.** 242 detected selective sweeps (20-kb) bins in Asian indicine compared to European taurine.

**Additional file 5: Table S4.** Detected selective sweeps in European taurine cattle based on F_ST_ and nucleotide diversity across Asian *Bos indicus* and European *Bos taurus* cattle (p.adj < 0.05) and their association with the closest genes.

**Additional file 6: Table S5.** Detected selective sweeps in Asian indicine cattle based on F_ST_ and nucleotide diversity across Asian *Bos indicus* and European *Bos taurus* cattle (p.adj < 0.05) and their association with the closest genes.

**Additional file 7: Table S6.** African taurine versus African indicine F_ST_ and nucleotide diversity in 20-kb overlapping windows with a 10-kb step-size.

**Additional file 8: Table S7.** 1194 detected selective sweeps (206 kb) bins in African taurine cattle compared to African indicine.

**Additional file 9: Table S8.** 324 detected selective sweeps (206 kb) bins in African indicine cattle compared to African taurine.

**Additional file 10: Table S9.** Detected Selective sweeps in African taurine cattle based on F_ST_ and nucleotide diversity across African *Bos indicus* and *Bos taurus* cattle (p.adj < 0.05) and their association with the closest genes.

**Additional file 11: Table S10.** detected selective sweeps in African indicine cattle based on Fst and nucleotide diversity across African *Bos indicus* and *Bos taurus* cattle (p.adj < 0.05) and their association with the closest genes.

**Additional file 12: Table S11.** Mouse phenotype enrichment for taurine cattle selective sweeps using GREAT [44].

**Additional file 13: Table S12.** Human phenotype enrichment for indicine cattle selective sweeps using GREAT [44].

**Additional file 14: Table S13.** Functional enrichment analysis, LOLA results for the European taurine selective sweeps.

**Additional file 15: Table S14.** Functional enrichment analysis, LOLA results for the Asian indicine selective sweeps.

**Additional file 16: Table S15.** Functional enrichment analysis, LOLA results for the African taurine selective sweeps.

**Additional file 17: Table S16.** Functional enrichment analysis, LOLA results for the African indicine selective sweeps.

**Additional file 18: Table S17.** Allele frequencies between European *Bos taurus* and Asian *Bos indicus* cattle for 23,494,872 SNPs with a MAF > 0.05.

**Additional file 19: Table S18.** Allele frequencies between African *Bos taurus* and *African Bos indicus* cattle for 22,943,179 SNPs with a MAF > 0.05.

**Additional file 20: Table S19.** M-values per genomic feature between European taurine and Asian indicine cattle.

**Additional file 21: Table S20.** M-values per genomic feature between African taurine and African indicine cattle.

**Additional file 22: Table S21.** Variant Effect Predictor results for fixed SNPs between taurine and indicine cattle ΔAF = 1 and 926 SNPs

**Additional file 23: Table S22.** Variant Effect Predictor results for fixed SNPs between taurine and indicine cattle ΔAF = 1, AND 476 SNPs.

**Additional file 24: Table S23.** rs447470311 (chr5:47726121) in 2907 imputed whole-genome sequences from run6 of the 1000 Bulls Genomes Project [24]..

**Additional file 25: Table S24.** Genotypes for 71 whole-genome sequences corresponding to 10 Brahman, 5 Africander, 56 Tropical composite animals for rs447470311 (chr5:47726121) and *HMGA2*-CNVR (chr5:48074233–48080443).

## Data Availability

All sequences were extracted from the 1000 Bull Genomes Project (Run6, March 2017) [24, 25]. All genotype data are fully accessible to readers partly through the 1000 Bull Genomes Consortium and partly through NCBI SRA (European taurine: PRJEB27309, PRJNA176557, PRJNA238491, PRJNA256210, PRJNA343262, and PRJNA474946; Asiatic indicine: PRJNA432125, PRJNA324822; African cattle, PRJEB1829, PRJNA312138). In addition, allele frequencies per population are available as Supplementary material via the permanent link to CSIRO’s Data Access Portal 10.25919/5ceb24e4ae2f8. Finally, F_ST_ and nucleotide diversity values are available in Additional file 2: Table S1.
