# Supplementary material for: Selection signatures in tropical cattle are enriched for promoter and coding regions and reveal missense mutations in the damage response gene HELB
Source: Genet Sel Evol. 2020 May 27;52:27. doi: 10.1186/s12711-020-00546-6 (PMC7251699; doi:10.1186/s12711-020-00546-6)
Supplement: Supplementary file 1 — Additional file 1. Figure S1. PCA of the genetic distance across all samples of European, Asian indicine, African taurine and African indicine cattle based on their genome (a) and (b) or breed of origin (c) and (d). (a) PC1 and PC2 explaining 84.02% and 11.60% of the variability, respectively; (b) PC1 versus PC2 (77.74% and 7.92% of the total variability for PC1 and PC2, respectively); and (c) and (d) PC1 versus PC2 and PC1 versus PC3 colour coded according to breed, respectively. Figure S2. PCA of the genetic distance to assess the clustering of sequences according to their breed of origin. (a) PC1 (77.74% of the total variability) and PC2 (7.92% of the total variability); and (b) PC1 versus PC3 (5.45% of total variability). Figure S3. Heterozygosity levels (number of heterozygous sites/total number of sites) in Asiatic, admixed and European taurine breeds classified according to genome of origin (i.e. orange = indicine, green = admixed, blue = taurine) (a) or to breed (b); and in African cattle classified according to genome of origin (c) or according to breed (d). Figure S4. Inbreeding coefficient, F, in Asiatic, admixed and European taurine breeds classified according to genome of origin (i.e. orange = indicine, green = admixed, blue = taurine) (a) or to breed (b); and in African cattle classified according to genome of origin (c) or to breed (d). Figure S5. Genetic variation and divergence in African cattle. (a) Proportion and number of private and shared SNPs in a set of African whole-genome sequences corresponding to 12 N’Dama (taurine in blue), 26 Uganda-mixed, 5 Africander and 10 Ankole (Sanga, zebu-taurine in green) and 10 Oganden and 10 Boran (Zebu or indicine in orange) (b) Nucleotide diversity was estimated in 20-kb genomic intervals for N’Dama π = 0.17%, Sanga π = 0.29% and indicine π = 0.32% sequences. Correlations between estimated reference allele frequencies (RAF) between taurine and indicine (c), Sanga and indicine (d) and Sanga and taurine (e). B [file 12711_2020_546_MOESM1_ESM.docx]

**Additional Material**

**Selection signatures in tropical cattle are enriched for promoter and coding regions and reveal missense mutations in the damage response gene *HELB***

Marina Naval-Sánchez^1#*^, Laercio R Porto-Neto^1^, Diercles F Cardoso^1,2##^, Ben J Hayes^3^, Hans D Daetwyler^4,5^, James Kijas^1^ and Antonio Reverter^1^

^1^CSIRO Agriculture & Food, 306 Carmody Rd., St. Lucia, Brisbane, QLD 4067, Australia

^2^Department of Animal Science, School of Agricultural and Veterinarian Sciences, Sao Paulo State University (UNESP), Jaboticabal, SP, Brazil

^3^Queensland Alliance for Agriculture and Food Innovation, The University of Queensland, St Lucia, Queensland 4067, Australia

^4^Agriculture Victoria, AgriBio, Centre for AgriBioscience, Bundoora, Victoria 3083, Australia

^5^School of Applied Systems Biology, La Trobe University, Bundoora, Victoria 3083, Australia

#Current address, Institute of Molecular Biosciences, The University of Queensland, 306 Carmody Road, St. Lucia, Brisbane, QLD 4067, Australia

##Current address, Centre for Genetic Improvement of Livestock, University of Guelph, 50 Stone Road East, Guelph, ON, N1G2W1, Canada,

*Corresponding address


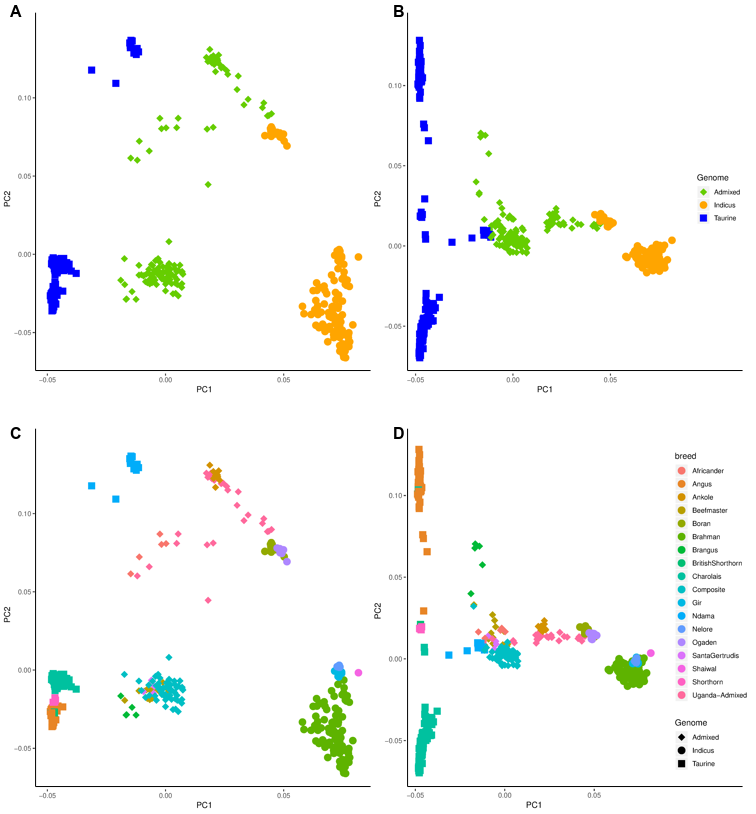


**Figure S1:**  **PCA of the genetic distance across all samples** of European, Asian indicine, African taurine and African indicine cattle based on their genome **(a)** and **(b)** or breed of origin **(c)** and **(d)**. **(a)** PC1 and PC2 explaining 84.02% and 11.60 % of the variability, respectively; **(b)** PC1 versus PC2 (77.74% and 7.92% of the total variability for PC1 and PC2, respectively); and **(c)** and **(d)** PC1 versus PC2 and PC1 versus PC3 colour coded according to breed, respectively.


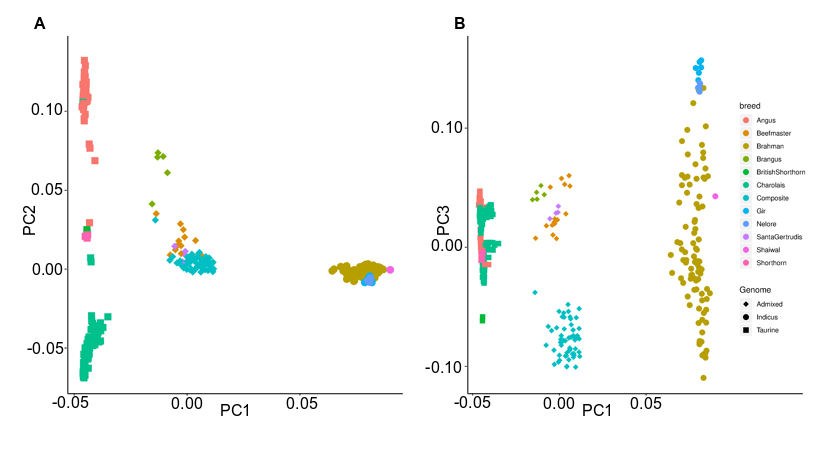


**Figure S2.** **PCA of the genetic distance to assess the clustering of sequences according to their breed of origin. (a)** PC1 (77.74% of the total variability) and PC2 (7.92% of the total variability); and **(b)** PC1 versus PC3 (5.45% of total variability).

**
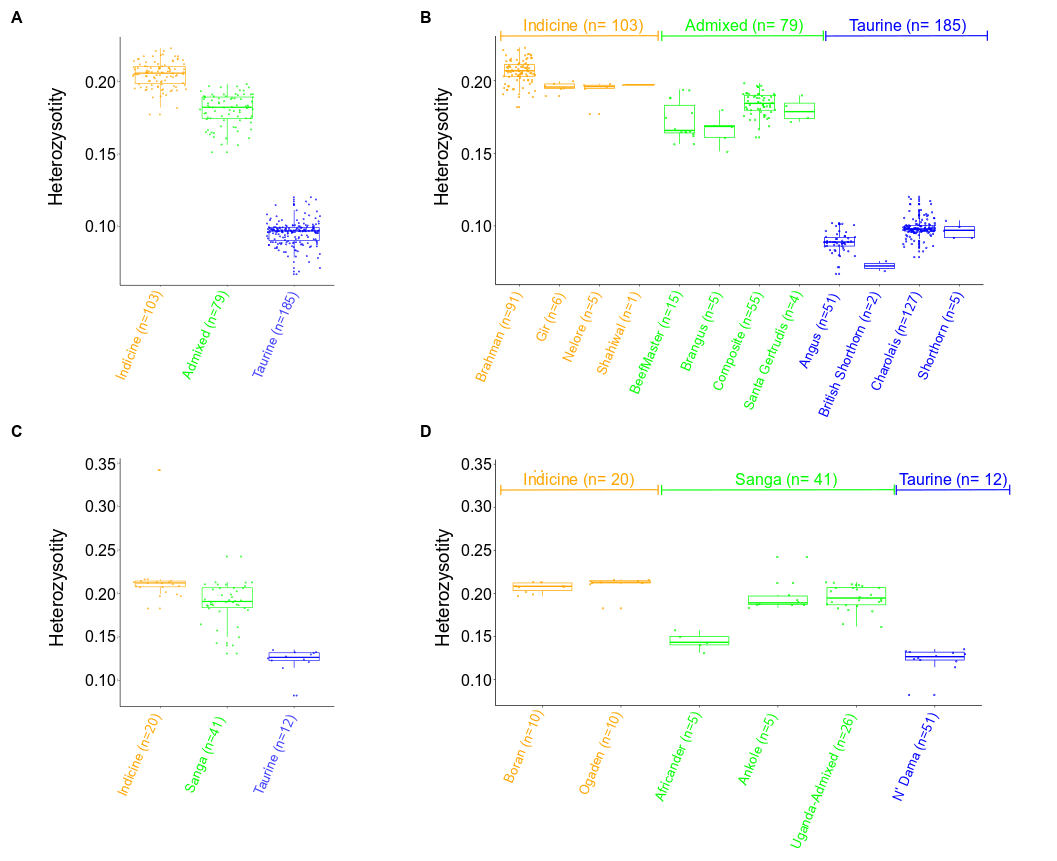
 Figure S3. Heterozygosity levels** (number of heterozygous sites/ total number of sites) in Asiatic, admixed and European taurine breeds classified according to genome of origin (i.e. orange = indicine, green = admixed, blue = taurine) **(a)** or to breed **(b)**; and in African cattle classified according to genome of origin **(c)** or according to breed **(d)**.


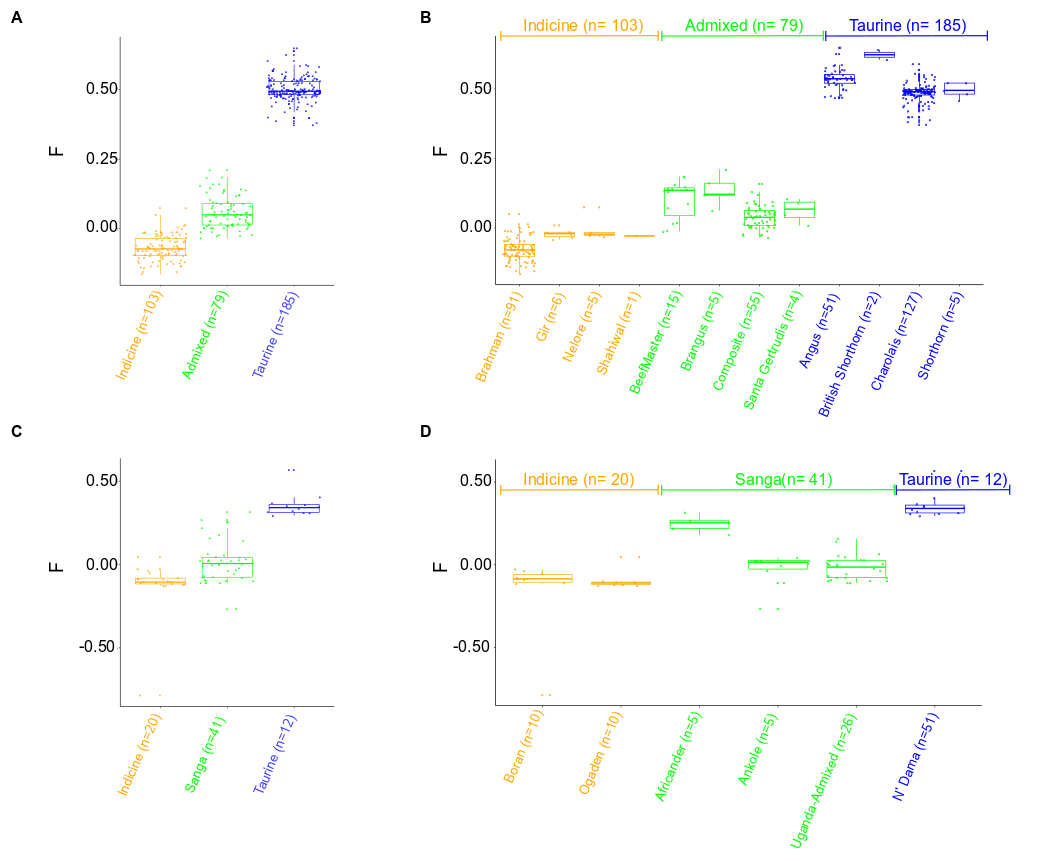


**Figure S4. Inbreeding coefficient, F,** in Asiatic, admixed and European taurine breeds classified according to genome of origin (i.e. orange = indicine, green = admixed, blue = taurine) **(a)** or to breed **(b)**; and in African cattle classified according to genome of origin **(c)** or to breed **(d).**


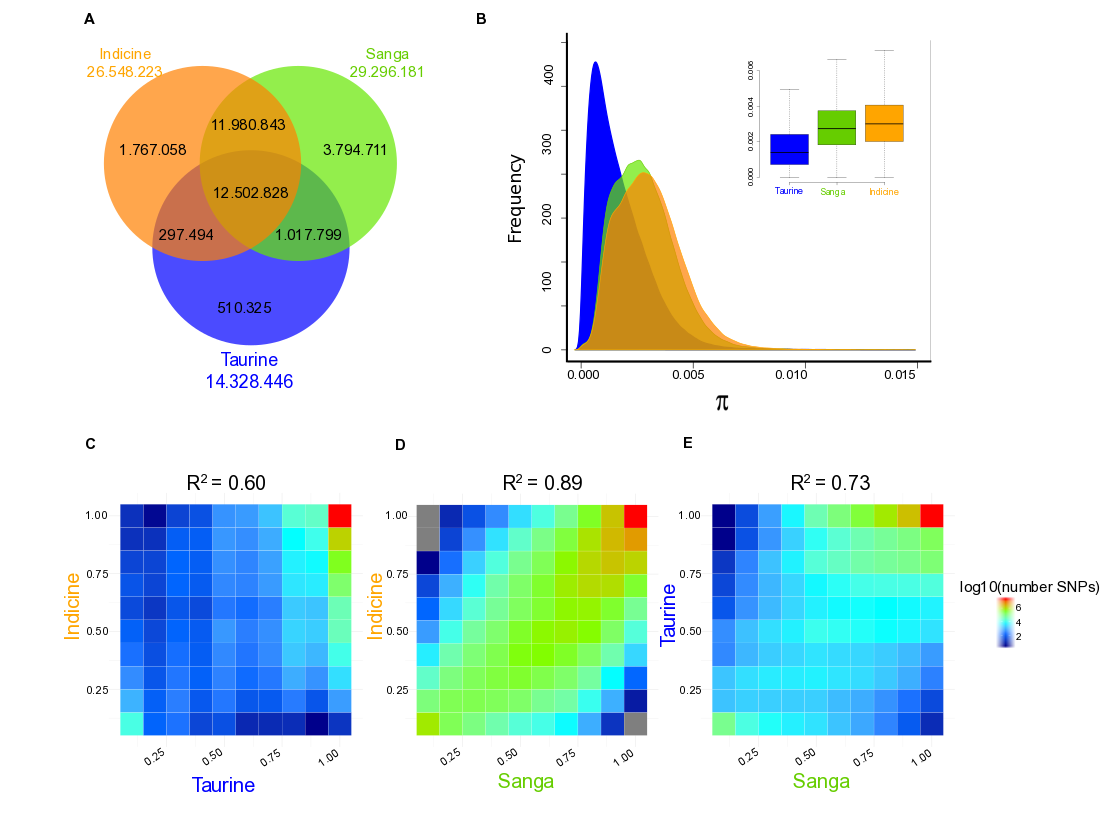


**Figure S5. Genetic variation and divergence in African cattle.** **(a)** Proportion and number of private and shared in a set of African whole-genome sequences corresponding to 12 N’Dama (taurine in blue), 26 Uganda-mixed, 5 Africander and 10 Ankole (Sanga, zebu-taurine in green) and 10 Oganden and 10 Boran (Zebu or indicine in orange) **(b)** Nucleotide diversity was estimated in 20-kb genomic intervals for N’Dama π = 0.17%, Sanga π = 0.29% and indicine π = 0.32% sequences. Correlations between estimated reference allele frequencies (RAF) between taurine and indicine **(c)**, Sanga and indicine **(d)** and Sanga and taurine **(e)**. Bins were estimated for each genome of origin or population, then compared between populations and visualised in heatmaps. The colors get warmer as the number of SNP counts increases.
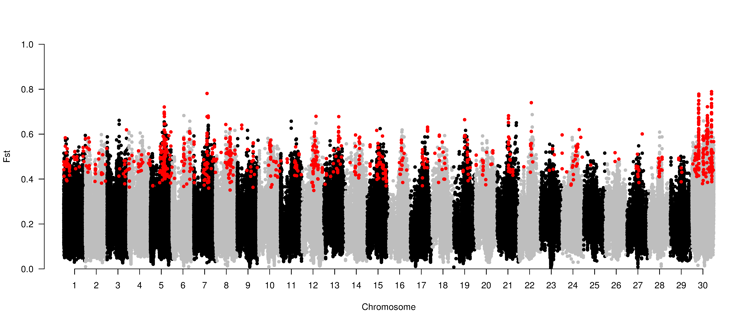


**Figure S6.** FST measure in 20-kb genome-wide overlapping bins with a 10 kb step size.

**
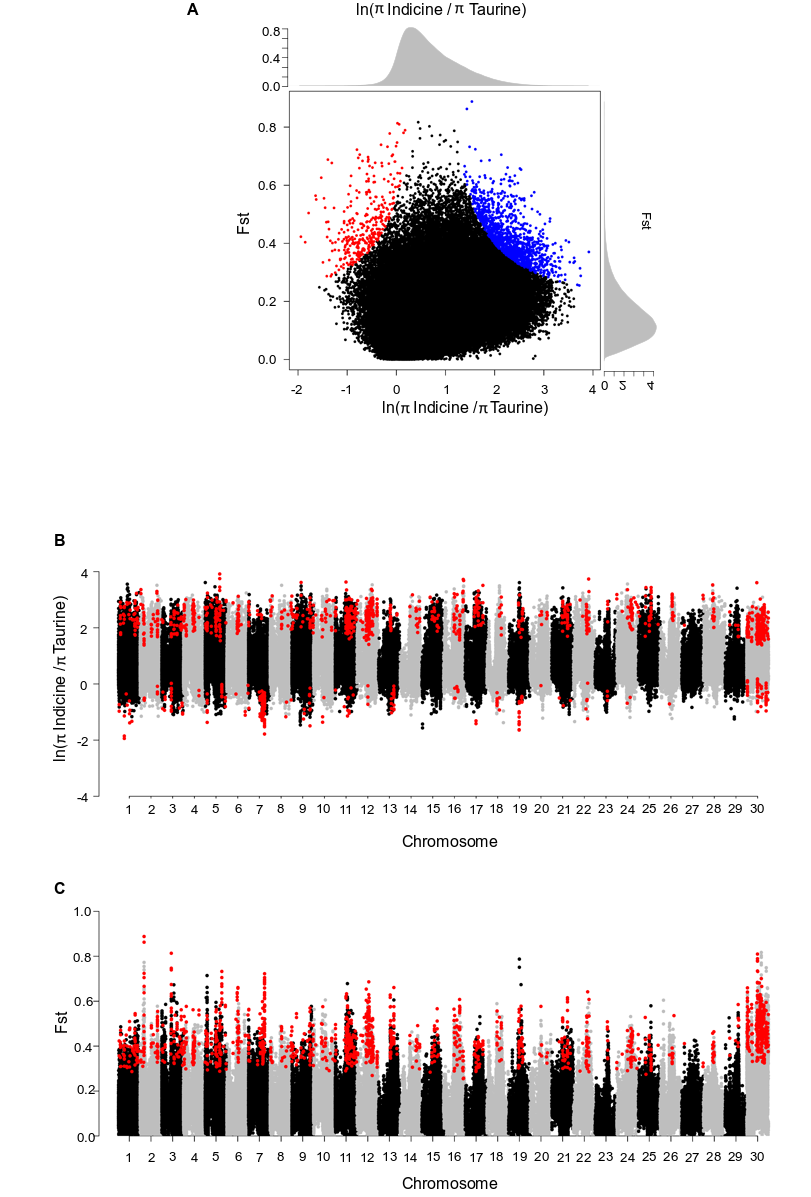
**

**Figure S7. Candidate selective sweeps in taurine and indicine in African cattle. (a)** Population differentiation (F_ST_) and relative nucleotide diversity between taurine and indicine cattle in genome-wide 20-kb genomic bins. **(b)** Genome-wide distribution of relative nucleotide diversity. Positive values represent candidate sweeps in taurine cattle and negative values in indicine. **(c)** F_ST_ measure in 20-kb genome-wide overlapping windows with a 10-kb step size.

**
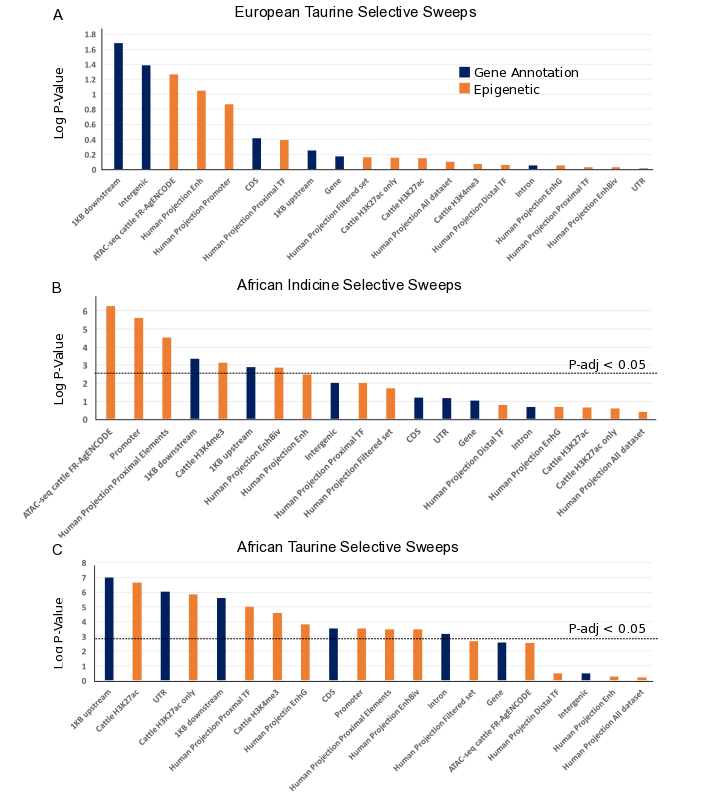
**

**Figure S8.** **Genomic feature enrichment in selective sweeps.** Strength of enrichment for 20 genomic features within 372 European taurine regions **(a)**; 611 African indicine regions **(b)**; 117 African taurine regions **(c)**.

**
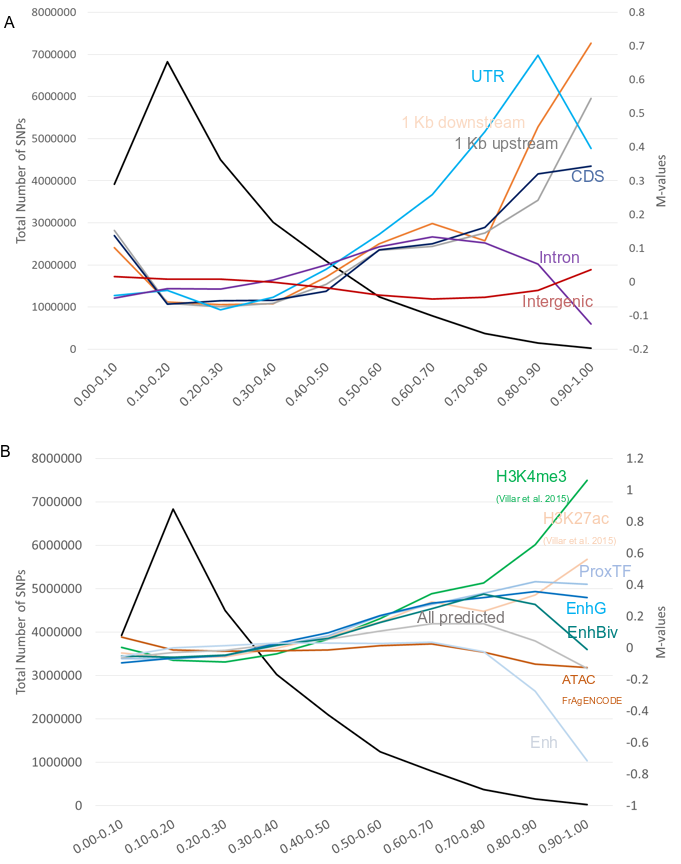
**

**Figure S9. Intersection of delta allele frequency (ΔAF) with different gene annotations.** **(a)** Genome annotation derived and **(b)** using predicted [20] and experimental annotations in cattle [48, 49].


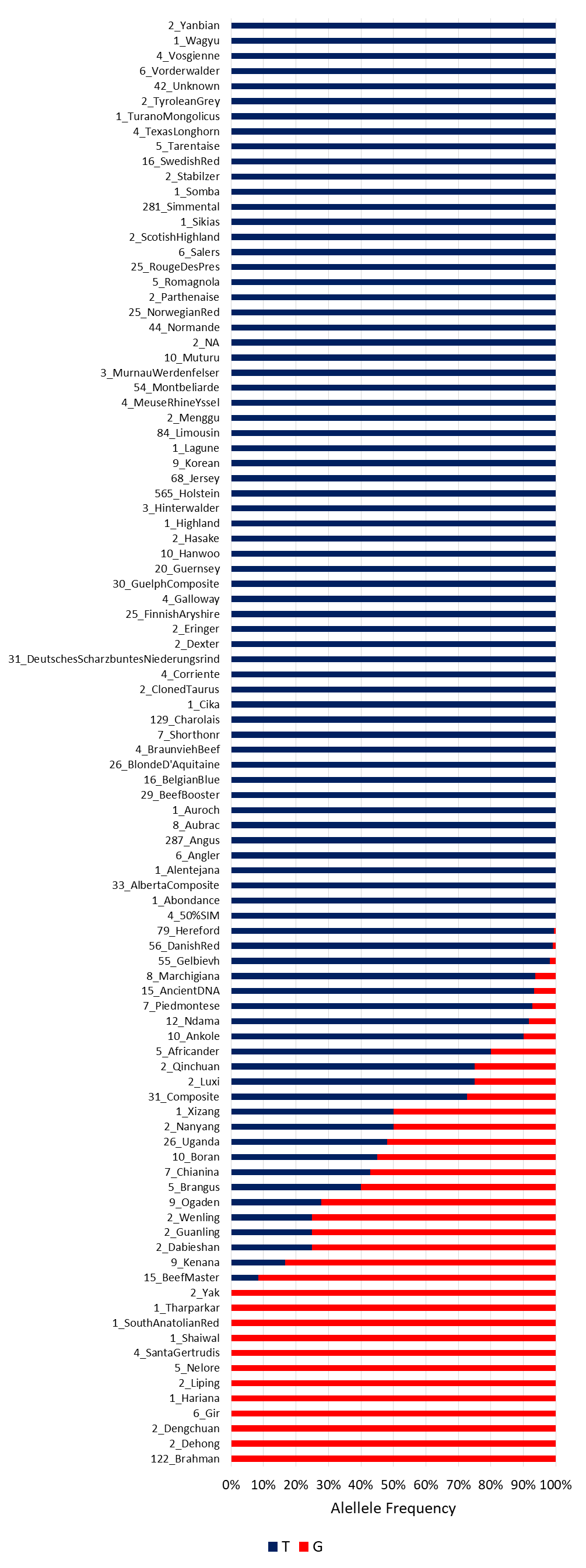


**Figure S10.** The 36 breeds in run6 of the 1000 Bull Genomes Project which present allele G at SNP rs447470311 (chr5:47726121) [24].

**
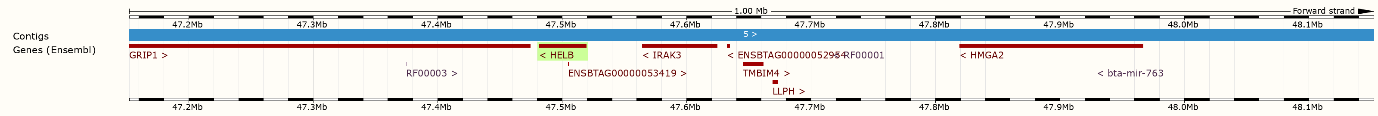

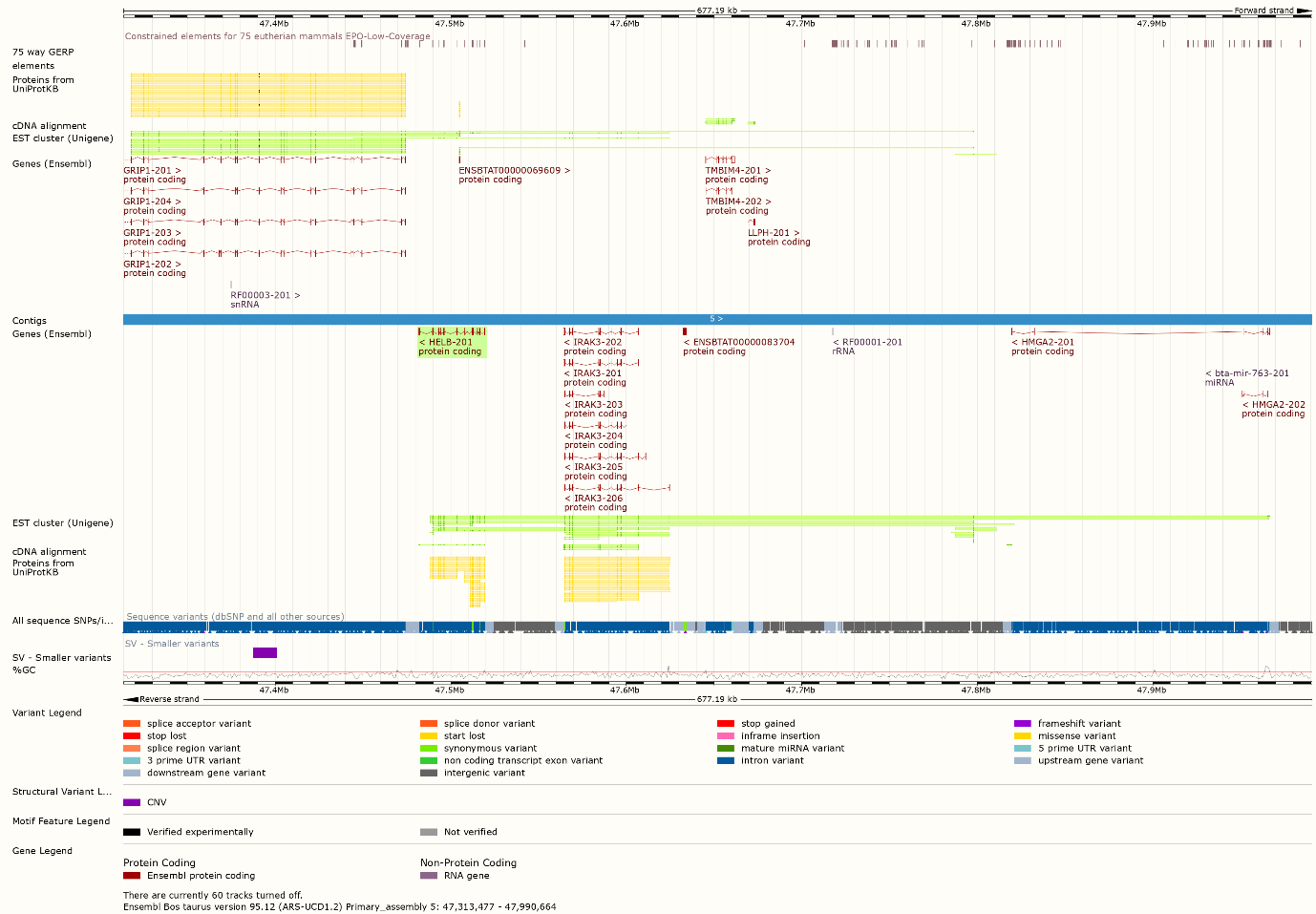
**

**Figure S11.** Regions that include selective sweeps in the cattle genome new assembly (ARS-UCD 1.2) at coordinates chr5: 47,481,051 – 47,520,235.
